# Research progress on epilepsy with myoclonic absence

**DOI:** 10.1186/s42494-025-00218-2

**Published:** 2025-05-16

**Authors:** Fen Tang, Minting Li, Liangmin Liu, Xuemei Wang, Bing Qin

**Affiliations:** Epilepsy Center, Department of Neurosurgery, The First Affiliated Hospital of Jinan University, Guangzhou, 510630 China

**Keywords:** Epilepsy with myoclonic absence, Genetic etiology, Pathophysiology

## Abstract

Epilepsy with myoclonic absence (EMA) is a rare childhood-onset generalized epilepsy syndrome characterized by myoclonic absence seizures. First discovered by Tassinari et al. in 1969, EMA has been extensively studied by researchers from all over the world. This review synthesizes recent studies on EMA, covering its discovery history, classification, epidemiology, pathophysiology, etiology, clinical manifestations, diagnosis and differential diagnosis, treatment, prognosis and evolution, and especially discusses the etiology and pathophysiology mechanism, to help clinicians understand this relatively rare epilepsy syndrome, reduce the rate of missed diagnosis and misdiagnosis, and effectively guide treatment to alleviate the long-term cognitive impairment in affected individuals.

## Background

Epilepsy with myoclonic absence (EMA) is a rare hereditary and heterogeneity generalized epilepsy syndrome originated from childhood, which is characterized by myoclonic absence (MA) seizures as the only or main seizures type. Interictal and ictal EEG shows 3 Hz generalized spike-wave. There is a strict and constant time-locked relationship between EEG and EMG during the ictal period [1, 2]. Functional imaging reveals that the cortical-thalamic loop may be involved in the genesis of MA [3]. In 2022, International League against Epilepsy (ILAE) classified EMA as a hereditary generalized epilepsy syndrome with childhood onset presentation [4].

## The history overview and classification evolution of EMA

In 1969, Italian epileptologist Carlo Alberto Tassinari first described the unique seizure type of myoclonic absence [5]. As the number of accumulated cases increased, Tassinari firstly named the syndrome as "epilepsy with myoclonic absence" in 1985, providing comprehensive clinical descriptions of its epidemiology, seizure types, EEG characteristics, evolution, diagnosis, and treatment. This is why the syndrome is also referred as "Tassinari syndrome" [1, 2].

In 1985, EMA was classified as idiopathic and/or symptomatic generalized epilepsy syndrome according to the classification of epilepsy and epilepsy syndrome of ILAE [6]. In 1989, EMA was categorized as cryptogenic or symptomatic generalized epilepsy syndrome [7]. In 2001, the ILAE Task Force on Classification and Terminology identified myoclonic absence seizures as an independent subtype of generalized seizures, while EMA was classified as an idiopathic generalized epilepsy syndrome [8]. In 2006, the report of the ILAE Classification Core Group recommended myoclonic absence seizure should be categorized as a subtype of generalized absence seizures and EMA should be classified as a childhood-onset epileptic syndrome [9]. In 2010, the report of the ILAE Commission on Classification and Terminology classified myoclonic absence seizure as a unique subtype of generalized absence seizures and EMA as an epileptic syndrome with childhood onset [10]. The position paper of the ILAE Commission for Classification and Terminology in 2017 categorized myoclonic absence seizure as a subtype seizure of non-motor absence seizures of generalized onset [11]. In 2022, the ILAE classification defined EMA, previously known as "Bureau-Tassinari syndrome", as a hereditary generalized epilepsy syndrome with childhood onset. It also clearly provided the mandatory, alert and exclusionary criteria for EMA [4].

## Epidemiology

EMA is a rare childhood-onset epilepsy syndrome, accounting for 0.5–1% of total epilepsy patients. The syndrome typically manifests between 6 months to 12.5 years of age, with a peak age around 7 years. A pronounced male predominance (70%) is observed. Approximately 20–25% of patients with EMA report a family history of epilepsy, typically generalized epilepsy. Furthermore, no cases of EMA with adult onset have been reported thus far [1, 2, 4].

## Pathophysiology

The pathophysiological mechanism of EMA remains unclear and has been rarely discussed in the literature. Despite both classic absence seizures and myoclonic absence seizures exhibiting a 3 Hz spike-wave discharge on EEG, a key unresolved question is why motor symptoms (e.g., myoclonus) are prominent in EMA but absent in typical absence epilepsy.

**Fig. 1:**
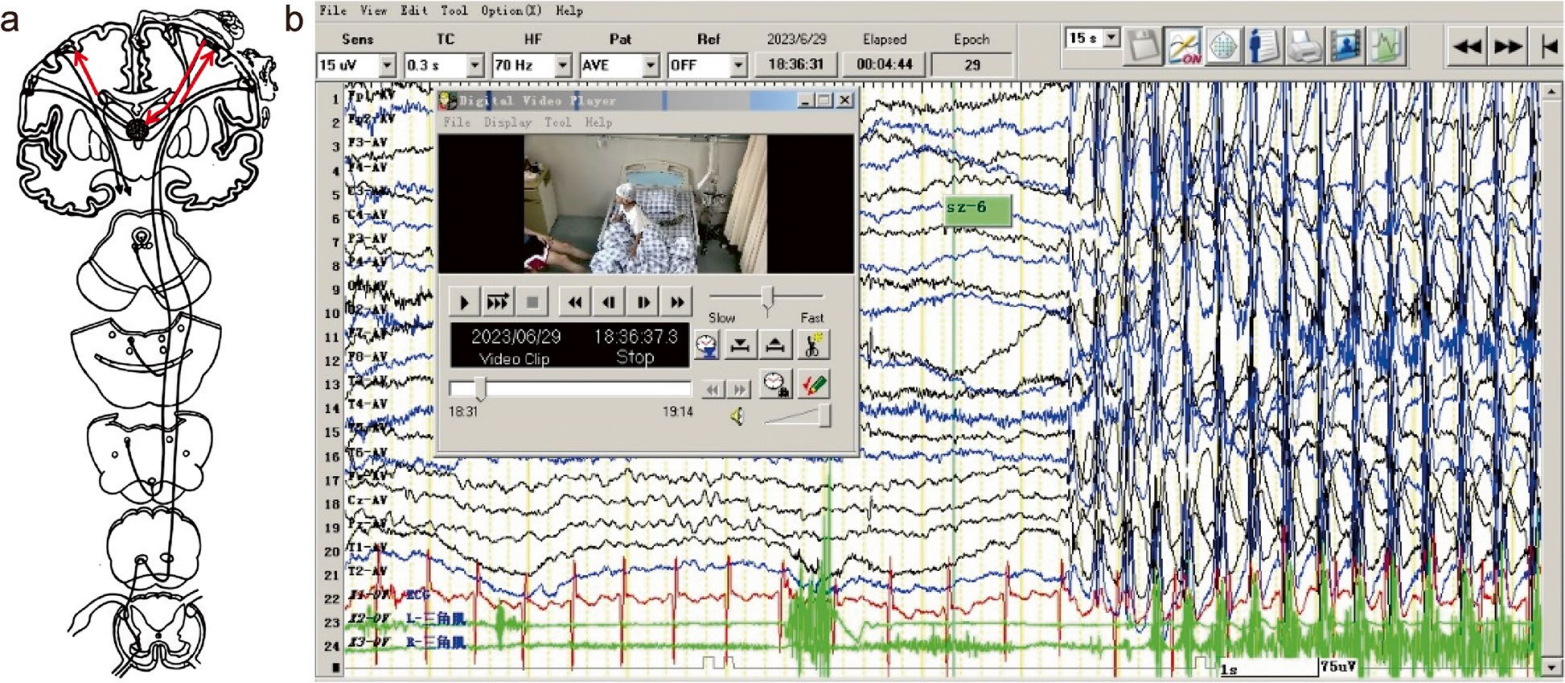
**a **Thalamocortical loop and the motor system of the cerebral cortex would be activated during myoclonic absence seizure. Permission was granted by Guerrini et al. (©AccScience Publishing [13]) to reuse this figure. **b **3Hz Generalized polyspike-waves time-locked with myoclonic jerks during myoclonic absence seizure

Previous experimental studies have confirmed that the activation of a neural loop—including the cerebral cortex, thalamic reticular nucleus and thalamus—produces the 3 Hz spike-slow wave in absence seizures. In vivo studies on synchronous EEG-functional magnetic resonance (EEG-fMRI) in childhood absence epilepsy showed that primary sensory (visual, auditory, somatosensory), motor (Rolandic) areas and frontoparietal association cortex were involved during absence seizures. The mechanisms underlying epileptic myoclonus (including cortical myoclonus, thalamocortical myoclonus and medulla reticular myoclonus) indicated that cerebral cortex, thalamus and medulla reticular were involved in the genesis of epileptic myoclonus [12, 13]. Given these observations, it is plausible that in EMA, the 3 Hz spike-waves generated by the thalamocortical loop likely excessively drive the motor cortex, especially the precentral gyrus, leading to simultaneous occurrence of myoclonus (Fig. 1).

Single photon emission computed tomography (SPECT) is an important tool for studying brain functional network in vivo. To understand the mechanism of myoclonic absence epilepsy, Ikeda et al. [3] conducted a study in 2017 using technetium-99 m-ethyl cysteinate dimer SPECT on two EMA patients to evaluate the changes of cerebral blood perfusion (CBF) during seizures. Researchers injected radionuclide tracers during interictal and ictal seizures respectively, which were confirmed by polygraphic recording. The results showed that in addition to the thalamus and basal ganglia, the motor area around Rolandic cortex was also involved in MA, and the blood perfusion in motor area may be affected by the oscillation of cortical-thalamic network, which clarified the pathophysiological mechanism of EMA to some extent.

## Etiologies

Overall, the etiology of EMA remains unclear and appears to be heterogeneous, categorized into idiopathic, symptomatic and cryptogenic forms. In idiopathic cases, 20–25% of EMA patients had family history of epilepsy [1], whereas symptomatic EMA is associated with factors including prematurity, perinatal damage, consanguineous marriage, and congenital hemiparesis.

The rapid development of next-generation sequencing technologies in recent years has made it possible to analyze the etiology of EMA at the molecular level, thereby enhancing accurate diagnosis, treatment, prognostic assessment, and genetic counseling. In the following section, the genetic mutations associated with EMA over the years are systematically summarized.

Elia et al. [14] found a genetic cause of trisomy 12p syndrome in a child with EMA, which was caused by a balanced translocation of chromosomes 8–12. In addition, it has been reported that EMA associated with chromosome abnormalities includes 2q13 microdeletions [15], maternal 15q11-13 deletions [16] and inverted replication of chromosome 15 [16].

Bahi-Buisson et al. [17] discovered a mutation in the glutamate dehydrogenase (*GDH*) gene (encoding glutamate dehydrogenase) in four members of a single EMA family. The mutation in the *GDH* gene enhanced glutamate dehydrogenase's function, increasing oxidative deamination of glutamate and elevating levels of α-ketoglutaric acid and ammonia. This increase in enzymatic reactions leads to an increase in tricarboxylic acid cycle activity and the ATP/ADP ratio in pancreas islet beta cells. The latter activates KATP channels, followed by cell depolarization and excessive insulin release. De novo gain-of-function mutations in *GDH* are the cause of hyperinsulinemia/hyperammonemia (HI/HA) syndrome, epilepsy, and mental retardation.

Gökben et al. [18] reported a Turkish patient with glucose transporter type 1 deficiency syndrome (GLUT1DS) presenting as EMA and identified a hot-spot mutation (R126C) in the *SLC2A1* gene. The *SLC2A1* gene, located at 1p35-31.3, is solute carrier family 2 (facilitated glucose transporter) member 1. It spans approximately 35 kb and comprises 10 exons and 9 introns, encoding a 492–amino acid protein with 12 transmembrane domains. Glucose transporter 1 (Glut1), mainly expressed in endothelial cells and astrocytes of the blood-brain barrier, facilitates glucose transport across the blood-brain barrier into astrocytes to provide energy for the brain. Glut1 is encoded by *SLC2A1* gene. The pathogenic mutations in *SLC2A1* impair GLUT1 function, leading to defective glucose uptake by astrocytes and consequently causing brain "energy disorder".

Klitten et al. [19] identified a de novo balanced translocation: t(6; 22) (p21.32; q11.21), in one patient with EMA, resulting in truncated mutations in the *SYNGAP1* gene. *SYNGAP1* located on 6p21.32, comprises 19 exons and follows an autosomal-dominant inheritance pattern. This gene encodes synaptic GTPase-activating activator protein 1, a key regulator of the NMDA receptor (N-methyl-D-aspartate receptor) that plays a critical role in neural development and glutaminergic neurotransmission pathway (essential for learning and memory). Its dysfunction can lead to cognitive impairment, epilepsy, and so on.

In a retrospective study of 10 patients with EMA, Carter et al. [20] also found a pathogenic *SYNGAP1* variant in one patient and a large deletion of 17p.13.3 in another. Additionally, Hiraide et al. [21] reported two patients with EMA harboring de novo pathogenic variants of *SETD1B* gene: c.386 T > G (p.Val129Gly) and c.5653 C > T (p.Arg1885 Trp). The *SETD1B* (*SET* domain containing 1B) gene encodes a histone H3 lysine 4 (H3K4) methyltransferase, which is involved in the epigenetic regulation of gene expression and chromatin structure. H3K4 trimethylase activity disorder caused by *SETD1B* gene mutation has been pathogenically linked to EMA. Furthermore, Frydson et al. [22] found *FOXP1* and *MBD5* gene mutations in a 7-year-old child with EMA. *FOXP1* and *MBD5* gene mutations were associated with EMA complicated with mental retardation, and *MBD5* gene mutations were associated with non-convulsive status epilepticus. This case later progressed to super refractory status epilepticus. Finally, Ogawa et al. [15] reported a case of EMA with 2q13 microdeletion (GRCh37:chr2:111,408,390–113098686), including protein-coding genes of *BUB1, ACOXL, BCL2L11, ANAPC1, MERTK, TMEM87B, FBLN7, ZC3H8,* and *ZC3H6.* Abnormal combination of the above genes may underlie epileptogenesis and neurodevelopmental disorders.

## Clinical manifestations

Myoclonic absence seizure is the core symptom of EMA. The severity of impaired consciousness is usually milder than that in childhood absence epilepsy (CAE), however, the seizure duration (ranging from 8–60 s) exceeds that of CAE and does not encompass the entire course of EEG discharge [23]. Rhythmic myoclonic seizures primarily affect the shoulder and limb muscles with rare eyelid involvement, but may occasionally engage the perioral muscle [1, 2]. Additionally, myoclonic absence seizures can manifest with complex gestural automatisms [24]. Seizures occur multiple times daily (ranging from several to dozens), often ceasing abruptly. The tonic element affecting both shoulders is often present in myoclonus, leading to rigid abduction and elevation of the upper limbs. In some cases, the tonic element is continuous with changes in respiratory rhythm or apnea, and sometimes urinary incontinence may occur. Asymmetrical myoclonus may occur, and falls are uncommon, but the body may sway forward or backward when standing. MA status is rare, only 1 of 36 patients with EMA reported by Bureau et al. met criteria for was MA status [1]. One-third of children with EMA exhibit myoclonic absence as the sole seizure type, and two-thirds may develop other seizure types (e.g., GTCS, typical absence seizures), which may precede or coincide with MA. If the above seizure types occur, it often indicates a poor prognosis and may be symptomatic EMA. MA can be induced by hyperventilation or awakening, and 14% of MA can be induced by intermittent photic stimulation (IPS) [1, 2].

## Diagnosis and differential diagnosis

The diagnosis of EMA relies on a comprehensive evaluation integrating medical history, seizure semiology, and polygraphic video-electroencephalogram (VEEG), with magnetic resonance imaging (MRI) serving to exclude structural etiologies. Medical history should include the patient's age, sex, developmental milestone, precipitating factors, age of first onset, core seizure type, related other seizure types, seizure frequency, seizure duration and family history [25]. Ictal seizure semiology with corresponding EEG and EMG should be recorded by polygraphic video-EEG, as well as interictal epileptiform discharges, sleep periods, and responses to hyperventilation, intermittent photic stimulation and fixation-off sensitivity. The background EEG activity of EMA is typically remains normal, while interictal recordings demonstrate generalized spike-wave or polyspike-waves predominance in the frontal area. During ictal episodes, particularly myoclonic absence seizures, the EEG shows rhythmic 3 Hz generalized spike-wave or polyspike-waves activity,. accompanied by characteristic EMG manifesting as bilateral synchronous and symmetrical rhythmic EMG bursts, superimposed with gradually increasing tonic potential, resulting in patients gradual lifting of both upper limbs. A strict time-locked relationship exists between EEG and EMG, making the analysis of electro-clinical symptoms with MA crucial for EMA diagnosis [1]. The sleep cycle in EMA patients is generally normal, and although focal or multifocal abnormal discharges may be detected in a small number of children with EMA, their presence does not exclude the diagnosis of EMA [26, 27].

It is challenging to distinguish EMA from idiopathic EMA, symptomatic EMA, CAE and Jeavons syndrome (Table 1) [4, 23, 26]. In generally, symptomatic EMA is often associated with abnormal neurological signs, abnormal background activity of EEG and structural abnormalities on brain MRI. Myoclonic absence seizures, particularly those less than 2.5 Hz spike-wave or other atypical absence seizures, can be seen in epileptic encephalopathy or chromosome abnormality syndrome.

**Table 1:** Differential diagnosis among idiopathic EMA, symptomatic EMA, CAE and Jeavons syndrome

	Idiopathic EMA	Symptomatic EMA	CAE	Jeavons syndrome
Age at onset	6 months−12.5 years	6 months−12.5 years	4−10 years	2−14 years
Sex ratio	males: females = 7: 3	males: females = 7: 3	males: females = 2.5−4: 6−7.5	males: females = 1: 2
Prevalence	0.5−1% of all epilepsies	0.5−1% of all epilepsies	0.4−0.7‰ of all persons	1.2−2.7% of all epilepsies
Family history	20−25%	20−25%	15−44%	25−83%
Seizure type	myoclonic absence seizures	myoclonic absence seizures, GTCS, clonic, atonic, typical absence seizures	typical absence seizures	eyelid myoclonia with/without absence, typical absence, GTCS, myoclonic seizures
Seizure frequency	several ten times per day	several ten times per day	daily seizure	daily seizure
Chromozomal anomalies/gene mutation	Trisomy 12p maternal deletion of 15q11-q13, 15q inversion—deletion, 2q13 microdeletion *GDH, SLC2A1, SYNGAP1, SETD1B, FOXP1, MBD5* gene	none	recurrent CNVs (e.g., 15q11.2, 15q13.3, and 16p13.11 microdeletion) *SLC2A1, GABRG2, GABRA1 g*ene [23]	*CHD2, NEXMIF104*/*KIAA2022, NAA10, SYNGAP1,* *RORB g*ene [28]
MRI	normal	abnormal	normal	normal
EEG	BG: normal, OIRDA is rare IID: 3 Hz GSWDs ID: 3 Hz GSWDs time-locked with myoclonic jerks	BG: abnormal, OIRDA is rare IID: frequent isolated spike or irregular SWs over the anterior regions ID: 3 Hz GSWDs time-locked with myoclonic jerks	BG: normal, OIRDA in 21–30% IID: awake: 2.5−4 Hz GSW asleep: polyspike and wave may be seen in drowsiness and sleep only ID: 3 Hz GSWDs	BG: normal, frequent OIRDA IID: 3−6 Hz rregular generalized polyspike-and-wave complexes ID: eye-closure and IPS induced 3 Hz GSWDs
ASMs	ETX, VPA, LTG	ETX, VPA, LTG	ETX, VPA, LTG	VPA, BZDs, LEV
Prognosis	eventually remit	myoclonic absences persist and may evolve into a cryptogenic or symptomatic epilepsy syndrome. intellectual disability may become evident with age multiple seizure types may indicate a more unfavorable prognosis	absence seizures disappear with age in more than 90%	often drug-resistant, requiring lifelong treatment 20% of patients develop eyelid myoclonic status epilepticus

Abbreviations: *ASMs* Antiseizure medications, *BG* Background, *BZDs* Benzodiazepines, *CAE* Childhood absence epilepsy, *EMA* Epilepsy with myoclonic absence, *EEG* Electroencephalogram, *ETX* Ethosuximide, *GSWDs* Generalized spike-and-wave discharges, *GTCS* Generalized tonic-clonic seizure, *LEV* Levetiracetam, *LTG* Lamotrigine, *MRI* Magnetic resonance imaging, *OIRDA* Occipital intermittent rhythmic delta activity, *ID* Ictal discharge, *IID* Interictal discharge, *IPS* Intermittent photic stimulation, *VPA* Valproic acid

## Treatment, prognosis and evolution

The management of EMA include antiseizure medications (ASMs), vagus nerve stimulation, ketogenic diet and surgical treatment. Currently, ASMs remain the primary treatment modality for controlling MA. The first-line ASMs are sodium valproate, ethosuximide, and lamotrigine, which can be used alone or in combination [20]. Second-line ASMs include levetiracetam, acetazolamide, zonisamide, topiramate, and lacosamide [20], while carbamazepine, phenytoin, vigabatrin, gabapentin, and tiagabine should be avoided due to their potential to exacerbate seizures [26]. Idiopathic EMA, accounting for about one-third of EMA cases and characterized exclusively by myoclonic absence seizures, the prognosis is relatively benign, often with eventual remission. Studies have shown that the combination of sodium valproate and ethosuximide is particularly effective [1]. By contrast, if there are other seizure types, such as symptomatic EMA, which accounts for about two-thirds of patients with EMA, especially combined with generalized tonic–clonic seizures, it often indicates a poor prognosis. If MA persists, it may evolve into a cryptogenic or symptomatic epilepsy syndrome, with higher risks of drug-resistantce EMA associated with frequent generalized tonic–clonic seizures may also evolve into Lennox-Gastaut syndrome (LGS) [1, 2]. Ito et al. reported a child with drug-resistant EMA combined with tonic–clonic seizures achieving complete seizure freedom following the addition of low-dose phenobarbital to a regimen of valproate and ethosuximide. This therapeutic success may be attributed to phenobarbital’s regulation on GABA_A_ receptors, increasing chloride ion influx and reducing the excitability of postsynaptic neurons [29]. Supporting these findings, a retrospective study of three children with EMA refractory to conventional ASMs demonstrated that adjunctive rufinamide (RUF) led to seizure freedom in two patients and a 50% seizure reduction in the third, indicating that the addition of RUF had a promising effect [30]. Palliative corpus callosotomy may serve as a viable therapeutic alternative for children with medically refractory EMA. In 2022, Emma et al. demonstrated favorable outcomes in two children with drug-resistant EMA who underwent this procedure: one patient who received a full corpus callosotomy achieved seizure freedom, while another underwent anterior two-thirds corpus callosotomy experienced a reduction more than 50% in seizure frequency (from 30 to 5–10 daily episodes), with complete resolution of tonic seizures [20]. In most children with drug-resistant EMA, the serverity of cognitive decline is proportional to the duration of intractable epilepsy. Cognitive impairment in these patients often manifests as behavioral disturbances, such as attention-deficit/hyperactivity disorder (ADHD), aggression, impulse-control disorder, and learning disabilities [25]. Although seizures may gradually diminish over time, the accompanying cognitive deficits frequently persist, and complete functional recovery is rarely achieved. [31].

The evolution of EMA covers the development of seizures, epilepsy syndrome and intelligence. The course of EMA mainly depends on the existence of GTCS, regardless of encephalopathic features (such as psychomotor retardation, hemiparesis and behavioral disorders) or treatment timing [1]. Seizure evolution refers to the fact that patients with EMA may continue experiencing MA attacks for up to 10 years after onset, with seizures typically subsiding after 4 years on average. Notably, EMA can evolve into LGS in some individuals. A follow-up study by Tassinari et al. demonstrated that among 28 patients with MA, 13 exhibited intellectual impairment before or at MA onset, while 15 initially showed normal intelligence—of these, 8 remained normal throughout the evolution but 7 developed significant mental deterioration during disease progression. Overall, 20 patients (including 13 with pre-existing and 7 with acquired impairment) ultimately exhibited cognitive deficits, presenting a markedly different neurodevelopmental trajectory compared to childhood absence epilepsy [32].

## Conclusions and future perspectives

As a rare hereditary epilepsy syndrome, EMA requires future investigation into its pathophysiological mechanisms through the integration macro-scale (e.g., functional neuroimaging, electrophysiology) and micro-scale (e.g., neural circuit) approaches. In the future, it is necessary to conduct international and domestic multi-center cohort studies, leveraging next-generation sequencing technologies to disclosure the genetic etiology and the mechanism underlying epileptogensis.

## Data Availability

Not applicable.

## Abbreviations

ASMs: Antiseizure medications
BG: Background
BZDs: Benzodiazepines
CAE: Childhood absence epilepsy
EMA: Epilepsy with myoclonic absence
EEG: Electroencephalogram
ETX: Ethosuximide
GSWDs: Generalized spike-and-wave discharges
GTCS: Generalized tonic–clonic seizure
LEV: Levetiracetam
LTG: Lamotrigine
MRI: Magnetic resonance imaging
OIRDA: Occipital intermittent rhythmic delta activity
ID: Ictal discharge
IID: Interictal discharge
IPS: Intermittent photic stimulation
VPA: Valproic acid
